# Evaluation of Hold-Up Volume Determination Methods and Markers in Hydrophilic Interaction Liquid Chromatography

**DOI:** 10.3390/molecules28031372

**Published:** 2023-02-01

**Authors:** Lídia Redón, Xavier Subirats, Martí Rosés

**Affiliations:** Institute of Biomedicine (IBUB) and Department of Chemical Engineering and Analytical Chemistry, University of Barcelona, Martí i Franquès 1–11, 08028 Barcelona, Spain

**Keywords:** HILIC, homologous series, hold-up volume, hold-up time, retention volume

## Abstract

Common methods for hold-up time and volume determination in Reversed-Phase Liquid Chromatography (RPLC) have been tested for Hydrophilic Interaction Liquid Chromatography (HILIC). A zwitterionic ZIC-HILIC column has been used for the testing. The pycnometric determination method, based on differences in column weight when filled with water or organic solvent, provides the overall volume of solvent inside the column. This includes the volume of eluent semi-sorbed on the packing of the column, which acts as the main stationary phase. The homologous series approach, based on the retention behavior of homologues in relation to their molecular volume, allows the determination of accurate hold-up volumes. However, the application of this method is time-consuming. In some cases, large neutral markers with poor dipolarity/polarizability and hydrogen bonding interactions can be used as hold-up volume markers. This is the case of dodecylbenzene and nonadecane-2-one in clearly HILIC behaving chromatographic systems, the use of decanophenone as a marker can be even extended to the boundary between HILIC and RPLC. The elution volume of the marker remains nearly unaffected by the concentration of ammonium acetate in the mobile phase up to 20 mM. The injection of pure solvents to produce minor base-line disturbance as hold-up markers is strongly discouraged, since solvent peaks are complex to interpret and depend on the ionic strength of the eluent.

## 1. Introduction

It is well known that an appropriate measurement of the hold-up volume (*V*_M_) in a HPLC column is essential for an accurate determination of the retention factor (*k*), defined in Equation (1) from the retention volume of the analyte (*V*_R_). The determination of retention factor is crucial for the description and prediction of analyte retention (system suitability, retention modelling…) and the derivation of thermodynamic quantities implied in the chromatographic process [1].

(1) $$k=\frac{V_{R}-V_{M}}{V_{M}}$$

There are different definitions of the hold-up volume and related terms, such as dead volume, void volume, solvent volume, and mobile phase volume. Much of the controversy comes from the fact that the stationary phase is usually preferentially solvated by some of the components in the eluent, more or less strongly sorbed on the surface of the packing material, and the presence of transition layers to the flowing mobile phase, which are difficult to assign to stationary or mobile phases due to the absence of a clear boundary between them. Different definitions come from the distinct assignment of the volume of these layers to stationary or mobile phase, and the different methods may give different values depending on the extent of the measurement of the volume of these layers. This problem is particularly important in Hydrophilic Interaction Liquid Chromatography (HILIC), as compared to Reversed-Phase Liquid Chromatography (RPLC), because of the complex nature of the intermediate transition layers between the column packing and the mobile phase created by partially, but preferentially, sorption or semi-sorption of water responsible of the HILIC retention mechanism.

HILIC is a complementary technique to RPLC for the analysis of polar and ionic compounds that are poorly retained in RPLC and tend to elute very close to the hold-up volume [2,3,4]. HILIC uses a polar stationary phase, such as in Normal-Phase Liquid Chromatography (NPLC), but the mobile phase consists of a mixture of water and an organic solvent (acetonitrile commonly), as in RPLC. HILIC mobile phases have a high content in the organic solvent (more than 70% of acetonitrile), and thus they are less polar than the stationary phase, favoring the retention of polar analytes by the stationary phase. In fact, the main HILIC retention mechanism is the partition of the analyte between the flowing mobile phase and the different sorbed or semi-sorbed solvent layers between the mobile phase and the surface of the column packing, being this latter doubtfully denominated stationary phase. This packing stationary phase (generally silica alone or functionalized with a polar group), rather than be involved in the partition process, may only show additional interactions with analyte by adsorption, ion exchange, or hydrogen bonding [2,3,4,5,6,7].

The polar nature of the HILIC packing stationary phases determines a high affinity for the most polar components of the hydroorganic eluent. Thus, in the organic solvent/water mixtures used as mobile phases in HILIC, water is preferentially sorbed on the surface of the polar packing stationary phase. Some authors [8,9] propose the existence of three solvent regions of different composition inside the HILIC column: (I) a rigid quasi-immobilized water layer at the packing surface; (II) a diffuse hydroorganic interface region, enriched in water, of reduced translational mobility between the water layer and the bulk mobile phase; and (III) the nominal flowing mobile phase. Since sorbed water is in dynamic equilibrium, there is not a clear separation between the three regions and most likely a gradient of water-rich solvent concentration and mobility is formed between the sorbent surface and the bulk mobile phase [2,8,9,10,11,12]. The layer in the surface of the packing stationary phase is mostly strongly sorbed water with a very reduced mobility, but water sorption decreases, and mobility increases in the consecutive transition layers approximating the composition and mobility of the flowing mobile phase. All these layers are labile and in dynamic equilibrium with the flowing mobile phase, but they have a variable reduced mobility in reference to the one of mobile phase. Thus, they act as stationary phase because a solute in these layers is delayed in reference to the flowing mobile phase. The behavior is comparable to the one of the charged micelles or microemulsions used as pseudo-stationary phases in micellar or microemulsion electrokinetic chromatography.

The term “hold-up volume” (*V*_M_) is preferred in this work to “void volume” (*V*_0_) to prevent confusion. According to IUPAC [13], “hold-up volume” is the volume of eluent required to elute a component, the concentration of which in the stationary phase is negligible compared to that in the mobile phase; whereas “void volume” (also called “interstitial volume”), is equivalent to “the interparticle volume of the column”, defined as “the volume occupied by the mobile phase between the particles in the packed section of a column. In liquid chromatography, the interparticle volume is equal to the mobile-phase hold-up volume (*V*_M_) in the ideal case, neglecting any extra-column volume”. Although this definition might be convenient in RPLC, in HILIC a portion of the eluent flowing inside the column is preferentially adsorbed on the surface of the packed section and, in fact, acts as HILIC stationary phase and not as mobile phase. Thus, in HILIC part of the interstitial volume is mobile phase, and part stationary phase.

Pycnometry, homologous series, unretained neutral markers (organic and inorganic compounds), and minor disturbance (solvent peak) are the main methods proposed for hold-up volume measurement. However, these different approaches usually lead to different hold-up volume values [1,14,15,16,17]. In a recent publication [18], McCalley compared the hold-up times obtained in several HILIC columns by pycnometry, homologous series, and toluene as hold-up time marker. He concluded that toluene, although it is soluble to some extent in the HILIC stationary phase, can be used as an approximate measure of the hold-up time for most routine measurements. However, it provides hold-up volumes slightly larger than those obtained by the homologous series method, which could be a more accurate method for detailed kinetic or thermodynamic studies. Pycnometry was not recommended at all, since it also includes the amount of solvent associated with the stationary phase.

The purpose of the present study is to discern the information provided by the different approaches to obtain the hold-up volume and related quantities in a HILIC column with acetonitrile/water and methanol/water mobile phases, and proposing the most rigorous and simple methods and markers to measure the hold-up volume (or time) in HILIC [17].

## 2. Results and Discussion

### 2.1. Pycnometry

Pycnometry, or weight difference method, is a popular technique for determining the hold-up volume of the column [1]. In the pycnometric method, the mass of the column is measured when it is sequentially filled with two solvents of sufficiently different densities. The weight of the column filled with the solvent (*w*_column_) is the sum of a constant contribution due to the weight of the column cylinder, endfittings, and the bonded phase and support (*w*_constant_), and the weight of the filling solvent, which depends on the volume of solvent (*V*_solvent_) inside the column and its density (*ρ*_solvent_) according to Equation (2):

(2) $$w_{\mathrm{column}}=w_{\mathrm{constant}}+V_{\mathrm{solvent}}\cdot \rho_{\mathrm{solvent}}$$

If the column is purged with two solvents of quite different densities (for instance, water and an organic solvent), the volume of the filling solvent can be determined from the difference in density and weight using Equation (3):

(3) $$V_{\mathrm{solvent}}=\frac{w_{\mathrm{column},\mathrm{water}}-w_{\mathrm{column},\mathrm{organic}}}{\rho_{\mathrm{water}}-\rho_{\mathrm{organic}}}$$

where *w*_column,water_ and *w*_column,organic_ are the weights of the same column after being consecutively equilibrated with water and organic solvent, and their corresponding densities are *ρ*_water_ and *ρ*_organic_, respectively.

Pycnometry provides the overall volume of solvent inside the column, which some authors consider as a measure of the hold-up volume. However, this method may be inadequate in case of significant preferential solvation of the stationary phase by one or more of the mobile phase components [1,19,20,21]. This concern seems to be especially pertinent in HILIC because of the preferential absorption of water by the column packing.

To test the method in HILIC, the volume of the filling solvent in a ZIC-HILIC column was determined using water and acetonitrile in the one hand, and water and methanol in the other. The *V*_solvent_ values obtained were 1.950 ± 0.016 mL for acetonitrile/water solvents pair and 1.946 ± 0.009 mL for methanol/water pair using the tabulated densities at 25 °C of 0.9971 g mL^−1^, 0.7766 g mL^−1^, and 0.7866 g mL^−1^ for water, acetonitrile, and methanol, respectively [22,23]. Practically the same volume was found for both solvent pairs in the zwitterionic functionalized silica column, in a similar way that the one measured by McCalley and Neue for their underivatized silica column [5].

### 2.2. Homologous Series LFER Method

The common homologous series method consists of plotting the log of the retention volume of the series members versus the homologue number [1] and extrapolating the linear plot obtained to the zeroth homologue. Alternatively, the hold-up volume can be estimated from the intercept of the linear regression.

A variation of the method derived from Linear Free Energy Relationships (LFER) models was proposed in an earlier study [24]. In the Abraham LFER model [25], the LFER variable (log *k* in chromatography [26,27,28,29,30,31,32,33,34,35,36,37]), is given as a linear combination of the solute-solvent interactions modeled by the solute descriptors accounting for dispersion forces (*E*), dipolarity/polarizability (*S*), hydrogen bond acidity (*A*), hydrogen bond basicity (*B*) and molecular volume (*V*) according to Equation (4):

(4) logk=c+e⋅E+s⋅S+a⋅A+b⋅B+v⋅V

where *c* is a non-solute dependent term accounting mainly for the chromatographic phase ratio, and *e*, *s*, *a*, *b*, and *v* the complimentary descriptors of the chromatographic system, all of them obtained by linear regression of the retention of a series of solutes against their solute descriptors. The Abraham descriptors *E*, *S*, *A*, and *B* for individual homologues in a series are almost constant and only *V* changes sequentially (linearly in fact) with the member number (number of -CH_2_- groups in the side alkyl chain). Given the constancy of some of the descriptors of the homologous members, combination of Equations (1) and (4) leads to Equation (5) [38]:

(5) $$V_{R}=V_{M}\left(1+r^{0}\cdot 10^{v\cdot V}\right)$$

being the fitted *r*^0^ a constant value giving a joint measure of the difference in dipolarity, polarizability, and hydrogen bond acidity and basicity of both mobile and stationary phases (*r*^0^ = 10*^c^*^+*e*·*E*+*s*·*S*+*a*·*A*+*b*·*B*^). Equation (5) allows the determination of hold-up volume (*V*_M_) by non-linear regression of the retention volumes (*V*_R_) of the homologous series members against their LFER descriptor volumes (*V*).

Since *V*_M_ and *v* values are expected to be independent of the specific homologous series used for the chromatographic system characterization, several homologous series can be altogether analyzed in the same fitting equation [35]:

(6) $$V_{R}=V_{M}(1+\sum_{i=1}^{n}\left(r_{i}^{0}\cdot f_{i})\cdot 10^{v\;\cdot V}\right)$$

where *n* is the number of homologous series included in the model, and *f_i_* are binary flag descriptors (1 or 0) used as independent variables in the fitting (i.e., for homologues of *i*-th series, *f_i_* = 1 and *f_i_*_≠1_ = 0). More precise and reliable results should be obtained from fittings to Equation (6), since homologues from different series cover a broader chemical space in terms of solute-solvent interactions.

We have selected for the present study a HILIC column with probably the most popular bonded phase, a permanent zwitterion (sulfobetaine) grafted on porous silica. ZIC-HILIC columns are being used in applications involving carbohydrates, metabolites, acids and bases, organic and inorganic ions, metal complexes, amino acids, peptides, and protein digests. 

Homologous series candidates for the determination of hold-up volume should exhibit a wide range of molecular volumes and low retention. Therefore, since hydrogen bonding interactions favor partition into the water-rich stationary phase, homologues should have small *A* and *B* descriptors, the closer to zero, the better. Additionally, for the sake of instrumental simplicity and economy, homologues should also be detected by UV absorbance. According to these criteria, the series selected for the study were *n*-alkyl benzenes, *n*-alkyl phenones and *n*-alkyl ketones. All of them lack hydrogen bond donor capabilities, phenones, and ketones show similar *B* values and benzenes the lowest ones (Table 1). Ketones, although their lower UV absorbance, provide the two smallest homologues of the three series, which might be an additional benefit in order to define the curvature (*v* coefficient) of the fitting.

The hold-up volumes obtained from fittings to Equation (6) are presented in Table 2. Hold-up volume decreases when increasing the water content in acetonitrile/water mobile phases probably because the more water in the mobile phase, the thicker the water-rich transition layers that act as stationary phase and thus the lower mobile phase volume. However, for methanol/water mobile phases, the hold-up volume is quite constant, likely because the high similarity between methanol and water keeps the volume of the water adsorbed more constant.

**Figure 1:**
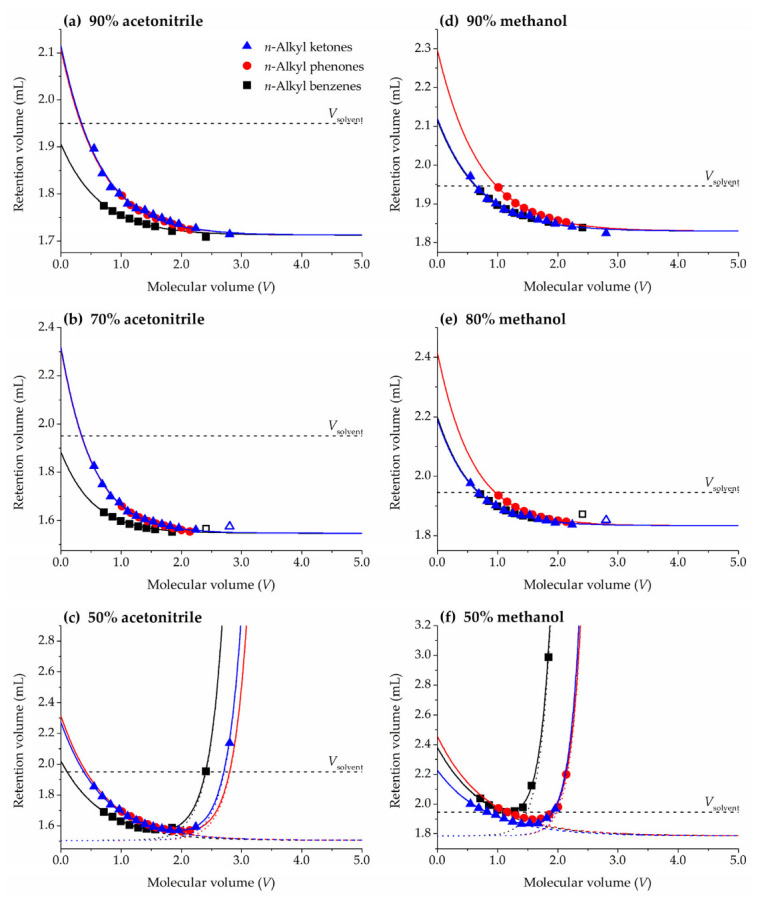
Representative plots of pure HILIC ((**a**) 90% acetonitrile, (**d**) 90% methanol), HILIC and slightly RPLC ((**b**) 70% acetonitrile, (**e**) 80% methanol), and mixed HILIC-RPLC ((**c**) 50% acetonitrile, (**f**) 50% methanol) behavior in the ZIC-HILIC column. For (**a**,**b**,**d**,**e**) solid lines represent fittings to Equation (6) (empty symbols in (**b**,**e**) represent homologues not included in the fittings). For (**c**,**f**) solid lines represent fittings to Supplementary Materials: Equation (S1), dashed and dotted lines show the contributions to the mixed mode of HILIC and RPLC, respectively. The dashed straight line shows the solvent volume inside the column pycnometrically measured (*V*_solvent_).

The fitted Abraham’s system coefficient *v* is negative (Table 2), since creation of a cavity in the more structured stationary phase (water-rich layers) will be easier for the smaller solutes, whereas largest solutes will tend to be solvated by the less structured mobile phase (hydroorganic eluent). Consequently, homologues retention in HILIC will decrease when the volume of the solute increases, as shown in Figure 1a,d. The contrary is expected for a RPLC mode, where the non-polar stationary phase is less structured than the hydroorganic mobile phase. This behavior is reflected with a positive value of *v*. Therefore, the sign of this coefficient allows us to distinguish between HILIC and RPLC behaviors in a particular system.

Figure 1 shows representative plots obtained with ZIC-HILIC column for some of the studied acetonitrile/water and methanol/water mobile phases. Retention of the members of the three homologous series decreases for mobile phases with a high content of organic solvent, acetonitrile (Figure 1a) or methanol (Figure 1d), which is typical of HILIC retention. By increasing the water content in the mobile phase, 70% of acetonitrile (Figure 1b) and 80% of methanol (Figure 1e), the HILIC column starts to show a mixed retention mechanism: mainly HILIC for the smallest homologues and mainly RPLC for the largest ones [35]. When the water content in the mobile phase is high enough, for instance 50% of acetonitrile (Figure 1c) and 50% of methanol (Figure 1f), both retention trends can be clearly observed and U-shape curves are obtained. In this work, we fill focus on mobile phase compositions with a typical HILIC behavior (readers are kindly referred to Supplementary Materials for a model adapted to the mixed HILIC-RPLC behavior).

The lowest retention in Figure 1 corresponds to the n-alkyl benzenes series for acetonitrile, but *n*-alkyl benzenes and *n*-alkyl ketones for methanol. Equation (5) relates retention to solute volume through system (*V*_M_ and *v*) and series (*r*^0^) parameters. *r*^0^ is the only term in Equation (5) depending on the chosen homologous series, since it includes the *E*, *S*, *A*, and *B* molecular descriptors that are common for all homologues belonging to the same series (Table 1). The least retained homologous series would be the one with the lowest *r*^0^ value. In fact, if *r*^0^ = 0 the series members would be not retained at all by the stationary phase (*V*_R_ = *V*_M_ in Equation (5)).

In HILIC systems with acetonitrile/water mixtures as eluents the main solute-solvent interactions responsible for retention are hydrogen-bonding related [34], since water-rich stationary phases have more significant hydrogen bonding features than acetonitrile-rich mobile phases. The three studied homologous series share a lack of hydrogen bond acidity (*A* = 0) and this interaction will not be further considered, but basicity needs to be evaluated. Actually, solute hydrogen bond acceptor capacity (*B*) is responsible for an increase in retention, and consequently the chromatographic retention of benzenes (*B* = 0.15) is lower than that of phenones and ketones (0.50 and 0.51, respectively). In fact, the fitted *r*^0^ values (Table S2) confirm that in general benzenes are less retained than phenones and ketones in HILIC mode with acetonitrile/water mobile phases. When methanol is used as organic modifier in the eluent in the ZIC-HILIC column studied in the present work, benzenes and ketones show similar *r*^0^ values and lower than those obtained for phenones. However, these results are more complex to interpret. To the best of our knowledge, only a few chromatographic systems using methanol have been characterized by means of the Abraham’s solvation parameter model [34,38], concluding that solute-solvent polarizability and dipolarity interactions (positive *e* and *s*) play a relevant role besides hydrogen-bonding (negative *b*). Thus, an increase in solutes *E* and *S* favors retention, whereas the contrary is observed for *B* (in contrast to acetonitrile/water which increase retention). The large *E* and *S* values of phenones (Table 1) leads to a larger retention of this series. Nevertheless, these results might be not representative of a general HILIC behavior using methanol as eluent.

### 2.3. Hold-Up Volume Markers

The injection of an “unretained” solute as a hold-up volume marker is the preferred and most widespread method among chromatographers because of its simplicity. The challenge is to find a truly unretained solute in HILIC, if it exists. The ideal marker must be small enough to access all the available mobile phase volume (interstitial + mesopores) and hydrophobic enough to stay out of the water-rich stationary phase. Here comes the first apparent contradiction, since in HILIC the smallest solutes are the most retained due to the higher cohesive nature of the stationary phase in relation to the hydroorganic mobile phase.

**Figure 2:**
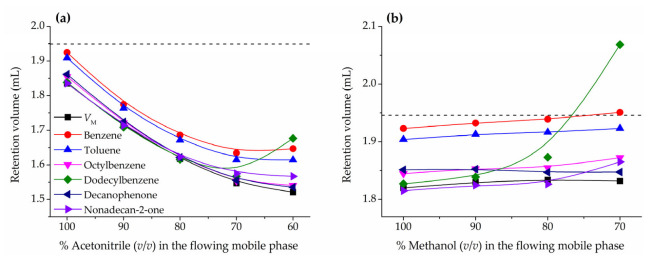
Variation of the retention for HILIC hold-up volume marker candidates with the composition of the mobile phase in the ZIC-HILIC column: (**a**) acetonitrile/water and (**b**) methanol/water mobile phases. The hold-up volume (*V*_M_) obtained from the homologous series approach (black squares) and the pycnometrically measured solvent volume (*V*_solvent_) (dashed straight line) are also plotted.

As already mentioned in the introduction, compounds such as benzene or toluene are commonly used as HILIC hold-up volume marker [5,11,18,40,41,42,43]. However, in a previous work it was found that these particular solutes are partially retained and larger solutes of the same type, such as octylbenzene and dodecylbenzene, should be more appropriate hold-up markers [24,35]. Figure 2a (acetonitrile) and Figure 2b (methanol) report the variation of the retention volumes of these markers with the mobile phase composition for acetonitrile/water and methanol/water mixtures, respectively, in the ZIC-HILIC column. Notice that in the plots are represented the mobile phase compositions corresponding to a HILIC behavior (down to 70% acetonitrile or 80% methanol) and the first percentage when mixed HILIC-RPLC starts to take place, which can be easily identified for an abrupt increase in the dodecylbenzene retention volume. For both organic solvents and eluent compositions typically used in HILIC applications, benzene and toluene are more retained than larger compounds, such as octylbenzene, dodecylbenzene, decanophenone, and nonadecan-2-one, which show similar retention volumes and in good agreement with the hold-up volume determined by the homologous series approach. These results are consistent with a HILIC retention mechanism based on the partition of solutes between the mobile phase and a hydroorganic stationary phase enriched in water partially immobilized on the chromatographic support. Benzene and toluene, although hydrophobic, are slightly soluble in water (1.8 and 0.5 g L^−1^ at 25 °C, respectively [44]), whereas the expected solubility of octylbenzene and dodecylbenzene is in the tens of µg L^−1^ range [45]. For any compound, since breaking water-water hydrogen bonding interactions to generate a cavity large enough to hold the solute is energy consuming, the higher the molecular volume, the lower the water solubility. Decanophenone and nonadecan-2-one are also insoluble in water, despite the contribution to polar surface area from oxygen atoms, with estimated solubilities not higher than 5 mg L^−1^ [45]. The tested compounds have molecular masses below 250 g mol^−1^ and roughly calculated volumes not exceeding 500 Å^3^. For instance, in the case of dodecylbenzene, a molecular volume of about 400 Å^3^ is compatible with a sphere of a diameter of about 9 Å or a more realistic cylinder with a length of 20 Å and a diameter of 5 Å [46]. These solute dimensions seem small enough to discard size exclusion effects in a column such the one used in this work with a porosity of 200 Å. However, the smallest benzenes due to their solubility in water might partition to some extent into the water-rich transition layers and therefore be retained by the stationary phase. Consequently, larger hydrophobic compounds appear to be a more convenient election for a hold-up volume marker in HILIC.

When the fraction of water is high enough to compromise the HILIC behavior of the system (<70% of acetonitrile and <80% of methanol) the retention of all the markers starts to significantly increase, especially dodecylbenzene. This is consistent with a RPLC behavior, in which the less cohesive bonded phase plays a relevant role in the partition process. Accordingly, large hydrophobic hold-up markers should be only used when the behave of the chromatographic system is clearly HILIC.

### 2.4. Minor Disturbance (Solvent Peak) Method

The injection of a pure solvent of a binary mobile phase produces a disturbance peak in the chromatogram that might be interpreted as the hold-up volume. However, this method is not straightforward since the selection of the injected pure eluent of the binary eluent (or even the injection solvent mixture) leads to significantly different disturbance peaks [1,47]. The application of the method in HILIC seems even trickier than in reversed- or normal-phase chromatography, since the injection of pure solvents surely affects in a complex way the diffuse hydroorganic interface of reduced mobility between the water layer sorbed on the bonded phase/support and the mobile phase.

**Figure 3:**
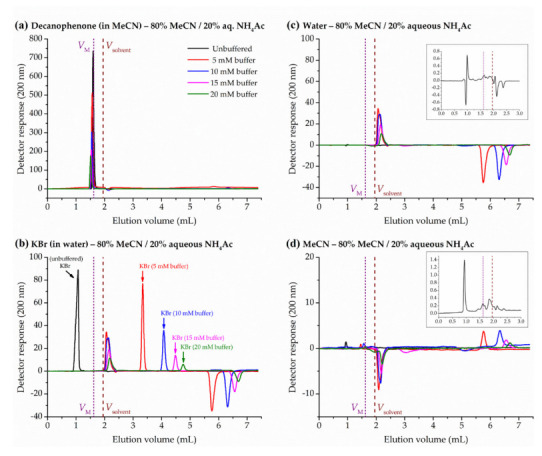
Effect of mobile phase ammonium acetate concentration (80% acetonitrile, MeCN) on the retention of hold-up marker candidates in the ZIC-HILIC column: (**a**) decanophenone, (**b**) potassium bromide, (**c**) water, and (**d**) acetonitrile. Dotted straight line corresponds to the hold-up volume from the homologous series approach (*V*_M_) and dashed straight line to the solvent volume pycnometrically measured (*V*_solvent_). Insets in (**c**,**d**) show the chromatograms corresponding to the injection of water and acetonitrile, respectively, in the unbuffered mobile phase.

In this work, pure water or acetonitrile were injected in acetonitrile/water (unbuffered) mobile phases in the HILIC range, leading to a first baseline disturbance at eluent volumes significantly lower than *V*_M_ measured by homologous series (about 1 mL), and a good number of diverse peaks around the total solvent volume pycnometrically determined. Insets in Figure 3c,d show representative peak disturbances obtained at 80% acetonitrile for the strong and weak solvent, water and acetonitrile, respectively. The volume of the first base line disturbance is clearly too low to be an accurate measure of the hold-up volume, and both solvents seem to be affected by some kind of exclusion from the total column porosity. This volume represents about the 50% of the total exchangeable solvent volume inside the column (*V*_solvent_, pycnometrically measured) and around the 60% of the expected mobile-phase volume (*V*_M_, determined by homologous series). These results might be consistent with the contribution of particle external porosity to the total column one reported in the literature for HILIC columns [48,49].

Anionic analytes are expected to be exposed to repulsive electrostatic interactions with the sulfonic group of the sulfobetaine bonded phase, and the positively charged quaternary ammonium group of the ligand is supposed to compensate the silanol activity [50]. Accordingly, the injection of an anionic analyte, such as bromide, in the ZIC-HILIC chromatographic system with an unbuffered eluent (with no salts) should lead to elution volumes lower than hold-up volume. Actually, the first solvent base line disturbance unexpectedly coincides with the elution volume of KBr (Figure 3b), suggesting that pure water or acetonitrile follow a similar path than bromide inside the column, probably in the frame of intraparticle porosity. In the last section of this paper, the effect of the salt concentration (ammonium acetate) in the eluent on KBr and solvent peaks is discussed.

### 2.5. Comparison of the Different Methods for the Hold-Up Volume Estimation

Figure 2 shows that hold-up volumes from the homologous series approach are clearly lower than the pycnometric *V*_solvent_ values because part of the solvent inside the column is acting as stationary phase sorbed on the column packing surface. The pycnometrically calculated *V*_solvent_ value of 1.950 ± 0.016 mL for acetonitrile and 1.946 ± 0.009 mL for methanol must contain the volume of mobile phase (*V*_M_) plus the stationary phase average volume of the water-rich layers semi-sorbed on the packing material. The results are similar to the ones previously obtained for different HILIC columns [35,51]. These findings show that the pycnometric method is useful to determine the volume of solvent inside the column, but it does not take into account the issue of a significant sorption of the mobile phase eluent on the column packing to become stationary phase. For acetonitrile/water mobile phases (Figure 2a) the difference between *V*_solvent_ and *V*_M_ is quite large, 0.2–0.4 mL with a maximum for 50% acetonitrile, but for methanol/water mixtures (Figure 2b) the difference is much smaller, only about 0.1 mL.

As shown in previous sections, the homologous series approach can provide better estimations of the hold-up volume than pycnometry. Conjoint analysis of retention of diverse homologous series by Equation (6) should be the most precise method for estimation of HILIC hold-up volumes, but it may be a long and tedious method. In routine work, it would be desirable to find an unretained marker with a retention volume as similar as possible to the hold-up volume from the homologous series approach. The performance of a marker to determine the hold-up volume (or time) can be directly measured from its retention factor, which is a direct measure of the relative error in *V*_M_ (Equation (1), being *V*_R_ the estimation of *V*_M_). The closer the *k* value to zero (*V*_R_ = *V*_M_), the better the candidate to unretained marker. The retention factors of the hold-up volume marker candidates have been measured using the *V*_M_ values determined from the homologous series approach (Table 2). Table 3 shows the retention factors of the three largest studied homologues of each series found for the ZIC-HILIC column for mobile phase compositions range exhibiting a clear HILIC behavior. Within each series the largest homologue shows the lowest retention factor, being those of dodecylbenzene and nonadecan-2-one the closest to zero, and therefore the recommended markers for hold-up volume estimation. Among these two compounds, dodecylbenzene has the additional benefit of a higher UV absorbance. Nevertheless, dodecylbenzene and nonadecan-2-one are more sensitive to the RPLC behavior when increasing the amount of water in the mobile phase, and at 70% acetonitrile or 80% methanol they are more retained than decanophenone.

### 2.6. Effects of the Salt Concentration on the Eluent

In HILIC, the concentration and nature of salts in the mobile phase play a significant role in the retention of ionic compounds [43,50,52,53]. Using ammonium formate and ammonium acetate in the 5–20 mM range in silica, zwitterionic, diol, and amide columns, McCalley and coworkers [52] described a general reduction in the retention of cations with the eluent ionic strength, but higher retention for anions. Interestingly, it was also reported this latter behavior for some neutral compounds, due to the effect of salt in the increment of the thickness of the water-rich stationary phase. In a different work conducted by Lucy and collaborators [43] exploring the effect of the electrolyte nature, similar conclusions were obtained from cations and anions, but the retention of the studied neutral analytes remained unaffected by salt concentration in the 1–20 mM range.

**Figure 4:**
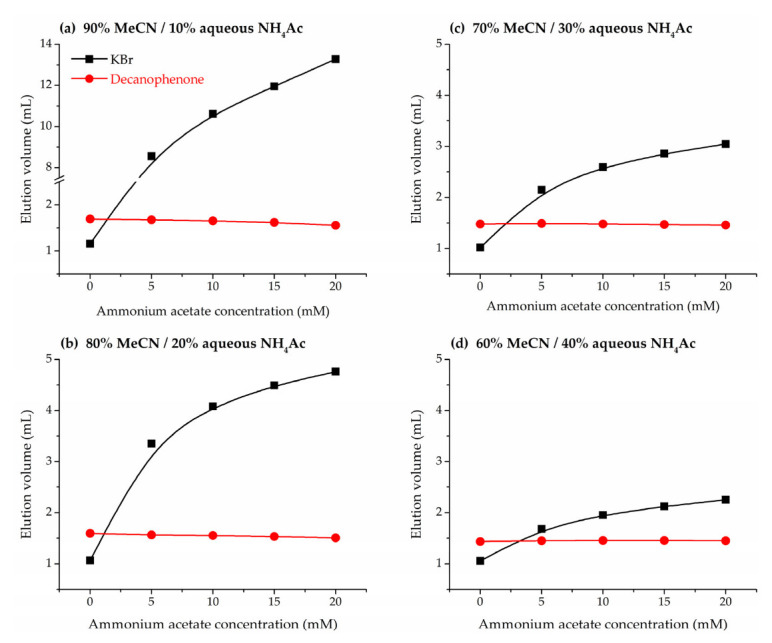
Variation of elution volumes of potassium bromide (black squares) and decanophenone (red dots) with the ammonium acetate concentration and the amount of acetonitrile (MeCN) in the mobile phase. Acetonitrile/aqueous buffer mixtures: (**a**) 90%, (**b**) 80%, (**c**) 70%, and (**d**) 60%.

Since the ionic strength of the eluent can play a role in the phase ratios of stationary and mobile phase, the following section of this work is devoted to the impact of the concentration of ammonium acetate buffer on the hold-up volume. To this purpose, retention volumes of the HILIC marker decanophenone were examined in acetonitrile/water mobile phases containing variable amounts of salt, in the range of concentrations in the eluent between 5 and 20 mM. As shown in Figure 2a, retention volumes of decanophenone are similar to the reference hold-up volumes determined by the homologous series approach in the boundary between HILIC and RPLC behavior, and thus it is a more reliable marker when it is intended to cover a wide range of mobile phase compositions. Potassium bromide was also injected as a positive control of the effect of ionic strength on the retention of ions. Figure 4 shows a nearly constant retention volume for dodecanophenone throughout the tested range, consistent with the hold-up volume determined by the homologous series approach in the absence of salt in the mobile phase. Therefore, hold-up volumes measured from neutral markers (including homologous series) seem to remain nearly unaffected by the concentration of ammonium acetate in the eluent.

In unbuffered acetonitrile/water mobile phases it has already shown that elution volumes of inorganic salts are lower than *V*_M_ and *V*_solvent_, and this was attributed to electrostatic repulsion between the inorganic salt anion and the sulfonate group of the zwitterionic bonded phase. Thus, the presence of buffer ions in the mobile phase might hinder this repulsion and lead to higher elution volumes. The positively charged quaternary ammonium of the zwitterion might be internally compensated by the acidic silanols of the support, making predominant the electrostatic effects of the negatively charged sulfonate group and allowing the stationary phase to behave as a weak cation exchanger [43,50]. In fact, Figure 4 shows that the retention volume of bromide ion increases with the ammonium acetate concentration in the mobile phase, and just a 5 mM concentration is enough to obtain retention volumes above *V*_M_ and *V*_solvent_. The increase in retention is especially relevant at 90% acetonitrile (Figure 4a), probably due to the accumulation of ions at the interface between mobile and stationary phases, reducing the electrostatic repulsion between with the sulphonate, and becomes less pronounced with the increase of the water proportion in the eluent.

As presented in Figure 3 and discussed in Section 2.4., the peak disturbance created upon the injection of a pure solvent (water or acetonitrile) is not a reliable measure of hold-up volume. Nevertheless, in the absence of salts in the eluent, the elution volume of bromide was similar to the first solvent peak of water and acetonitrile. Since the retention of bromide depends on the ionic strength of the mobile phase, we decided to investigate the effect of ammonium acetate on the solvent peaks. Now, the first peak is observed just after *V*_solvent_ (positive in the case of water, Figure 3c; negative for acetonitrile, Figure 3d) and a second peak beyond 5.5 mL (negative and positive, respectively). Whereas retention volumes of the first peak are nearly constant, the second one shifts to higher volumes with the concentration of buffer in the eluent. Interestingly, the peak areas corresponding to 5 and 10 mM are similar, and then the area is progressively reduced at 15 and 20 mM. This might be possibly related to the ion exchange properties of the zwitterionic bonded phase employed in this study [54]. In any case, in the presence of buffer in the mobile phase, the first disturbance peak appears beyond the *V*_solvent_, and therefore this method is definitely not suitable for the estimation of hold-up volumes.

## 3. Materials and Methods

### 3.1. Instrumentation

The HPLC system consisted of two LC-20AD pumps, an SIL-10AC autosampler, an SPD-10AVvp UV-Vis detector, and a CTO-10ASvp oven set at 25 °C, all from Shimadzu (Kyoto, Japan). The system was controlled by LC Solutions software from Shimadzu. The overall extra-column volume was subtracted from all the measured gross retention volumes.

The column employed was a ZIC-HILIC from Merck (Darmstadt, Germany), 5 µm, 150 mm × 4.6 mm, 200 Å pore size. The ZIC-HILIC column has a zwitterionic bonded phase (sulfobetaine) covalently attached to porous silica.

For pycnometric measurements an AT 261 DR analytical balance from Mettler-Toledo (Columbus, OH, USA) was used. The balance was located in a climatized room (22 ± 2 °C, 50 ± 5% humidity) and yearly calibrated by an accredited calibration laboratory (Mettler-Toledo, Barcelona, Spain).

### 3.2. Methods and Chromatographic Conditions

Pycnometric measurements were performed equilibrating the column with water, acetonitrile, and methanol during one hour at a flow rate of 0.5 mL min^−1^. After purging, the column was immediately capped with its corresponding endfittings and weighed in the calibrated analytical balance. All measurements were performed in triplicate.

The injection volume and the mobile phase flow rate were 1 µL and 0.5 mL min^−1^, respectively. Mobile phases consist of acetonitrile/water and methanol/water mixtures wherein the % in volume of organic solvent was varied between 100%, 90%, 80%, 70%, 60%, and 50%. The column was equilibrated for at least 20 min when changing the eluent composition. All injections were performed at least in duplicate. The three homologous series (*n*-alkyl benzenes, *n*-alkyl phenones, and *n*-alkyl ketones) were injected sequentially.

Detector wavelength was set at 210 nm for *n*-alkyl benzenes, 245 nm for *n*-alkyl phenones, and 275 nm for *n*-alkyl ketones. The hold-up volume markers candidates were measured at 254 nm and for the minor disturbance method the signal of water and acetonitrile was detected at 200 nm.

Extra-column volume was measured by the injection of 0.5 mg mL^−1^ aqueous solution of potassium bromide (Baker, Sanford, ME, USA, >99%), removing the column and connecting the injector directly to the detector, using water and different acetonitrile/water and methanol/water mixtures as eluents.

The flow rate (mL/min) was used to transform the elution times (min) recorded from chromatograms into volumes (mL), in order to facilitate the comparison with the hold-up volumes obtained from the different approaches considered in this work.

### 3.3. Chemicals and Solvents

The injected series of homologues (*n*-alkyl benzenes, *n*-alkyl phenones, and *n*-alkyl ketones) and the hold-up volume markers candidates (acetone, urea, dimethyl sulfoxide (DMSO), *N*,*N*-dimethylformamide (DMF), formamide, thiourea, uracil, potassium bromide, and lithium nitrate) were purchased from Acros Organics (Geel, Belgium), Alfa Aesar (Ward Hill, MA, USA), Baker, Carlo Erba (Emmendingen, Germany), Fluka (Buchs, Switzerland), Merck, Prolabo (Sion, Switzerland), and Sigma-Aldrich (St. Louis, MO, USA), all of high purity grade (≥97%). Stock solutions of the injected analytes were prepared in methanol at a concentration of 5 mg mL^−1^ except for inorganic salts, which were dissolved in water. *n*-Alkyl ketones were injected at stock solution concentration because of their low UV-Vis absorbance, but the rest of the analytes were diluted to 0.5 mg mL^−1^ before injection.

Water was obtained from a Milli-Q plus system from Millipore (Billerica, MA, USA) with a resistivity of 18.2 MΩ cm. Acetonitrile and methanol were HPLC gradient grade and from Labkem (Dublin, Ireland) and Chem-Lab (Zedelgem, Belgium).

Ammonium acetate (Sigma-Aldrich, >98%) was used in the evaluation of the effect of salt concentration in acetonitrile/water mobile phases, in a range between 5 and 20 mM.

### 3.4. Calculation

All calculations were done in MS Excel. Non-linear regressions were performed by the Solver tool and the statistics of the fittings were calculated through the Excel macro “Ref_GN_LM”, which is based on the Levenberg-Marquardt modification of the Gauss-Newton non-linear least-squares iterative algorithm [55].

## 4. Conclusions

The application of the common hold-up volume (or time) determination methods to HILIC columns is more problematic than to the classical RPLC columns and provides different information. Pycnometry with pure solvents (water, acetonitrile, methanol) provides the overall amount of eluent inside the column (*V*_solvent_). In HILIC mobile phases, *V*_solvent_ volume includes the hold-up volume or volume of flowing mobile phase (*V*_M_) and the volume of the water-rich transition layers adsorbed on the packing material, which in fact is the volume of the main stationary phase.

The homologous series method provides the hold-up volume by extrapolation of the retention of the homologues against their molecular volume. The extrapolated *V*_M_ values are significantly lower than the *V*_solvent_ values in the acetonitrile-rich mobile phases, because of the preferential sorption of water in the stationary phase, and slightly higher in the water-rich mobile phases. In addition, the method allows to differentiate between two different behaviors, HILIC or RPLC. In HILIC conditions, the retention of the homologues decreases when their volume increases (small solutes are more retained than large solutes in the water-rich stationary phase more polar than the mobile phase) showing a HILIC type stationary phase more structured than the mobile phase. When the water content in mobile phase increases (RPLC conditions) it is the contrary, retention increases with the solute volume.

In some cases, neutral markers can be a good alternative to homologous series for hold-up volume estimation, provided that they are carefully selected to exhibit negligible partition into the stationary phase. Hold-up volume markers in HILIC must be large and with poor dipolarity/polarizability and hydrogen bonding abilities, which is the case of dodecylbenzene, nonadecane-2-one or decanophenone. The first two markers are particularly suitable for pure HILIC behavior (e.g., >80% acetonitrile); the last one is recommendable when working in the boundary between HILIC and RPLC. The retention volume of decanophenone remains nearly unchanged in the range in mobile phases containing up to 20 mM of ammonium acetate.

The minor disturbance peaks produced by the injection of pure solvents are not suitable for the hold-up volume estimation, since they are complex to interpret and depend on the mobile phase buffer concentration.

## Figures and Tables

**Table 1:** Molecular descriptors of the homologous series (mean values for *E*, *S*, *A*, and *B*; ± standard deviation; range for *V*) considered in this work [39].

	*E*	*S*	*A*	*B*	*V*
**Homologous series**					
*n*-Alkyl benzenes	0.59 ± 0.01	0.50 ± 0.02	0.00 ± 0.00	0.15 ± 0.00	0.72–2.41
*n*-Alkyl phenones	0.78 ± 0.02	0.96 ± 0.02	0.00 ± 0.00	0.50 ± 0.01	1.01–2.14
*n*-Alkyl ketones	0.12 ± 0.03	0.68 ± 0.01	0.00 ± 0.01	0.51 ± 0.01	0.55–2.80

Molecular descriptors for individual homologues can be found in Supplementary Materials: Table S1.

**Table 2:** Hold-up volumes and *v* coefficients (±standard deviation) of the ZIC-HILIC column at the different mobile phase compositions (HILIC behavior) obtained from the fittings of retention to Equation (6).

*ϕ*_org_ (*v*/*v*)	*V*_M_ (mL)	*v*	*N*	*R* ^2^_adj_	*RMSE*
**Acetonitrile**					
100%	1.835 ± 0.004	−0.56 ± 0.05	31	0.982	0.004
90%	1.713 ± 0.003	−0.67 ± 0.04	31	0.985	0.004
80%	1.617 ± 0.002	−0.83 ± 0.03	31	0.993	0.004
70%	1.547 ± 0.002	−0.82 ± 0.03	29	0.996	0.003
**Methanol**					
100%	1.820 ± 0.004	−0.59 ± 0.04	31	0.985	0.004
90%	1.830 ± 0.003	−0.62 ± 0.04	31	0.987	0.004
80%	1.834 ± 0.003	−0.75 ± 0.04	29	0.993	0.003

*r*^0^ values, together with fittings to mixed HILIC-RPLC behavior, can be found in Table S2.

**Table 3:** Measured retention factors (*k*) of hold-up volume marker candidates for the ZIC-HILIC column.

Marker Candidate	Acetonitrile				Methanol		
	100%	90%	80%	70%	100%	90%	80%
***n*-Alkyl benzenes**							
Hexylbenzene	0.017	0.010	0.007	0.009	0.020	0.018	0.015
Octylbenzene	0.011	0.005	0.003	0.004	0.014	0.013	0.011
Dodecylbenzene	0.002	−0.003	−0.001	0.013	0.004	0.005	0.021
***n-Alkyl phenones***							
Octanophenone	0.021	0.013	0.010	0.014	0.024	0.018	0.012
Nonanophenone	0.017	0.009	0.006	0.009	0.020	0.015	0.009
Decanophenone	0.014	0.007	0.003	0.005	0.017	0.012	0.007
***n*-Alkyl ketones**							
Tridecan-2-one	0.016	0.013	0.011	0.014	0.012	0.010	0.005
Pentadecan-2-one	0.010	0.008	0.006	0.009	0.007	0.006	0.002
Nonadecan-2-one	0.000	0.001	0.004	0.018	−0.003	−0.003	0.010

## Data Availability

The data presented in this study are available on request from the authors.

## Supplementary Materials

The following supporting information can be downloaded at: https://www.mdpi.com/article/10.3390/molecules28031372/s1, Equations (S1) and (S2): Simultaneous contribution of HILIC and RPLC retention mechanisms; Table S1: Molecular descriptors of the homologues considered in this work; Table S2: *V*_M_, *v*, and *r*^0^ fitted values from homologous series retention volumes.

## Sample Availability

Samples of the compounds are not available from the authors.
