# Supplementary material for: Evaluation of Hold-Up Volume Determination Methods and Markers in Hydrophilic Interaction Liquid Chromatography
Source: Molecules. 2023 Feb 1;28(3):1372. doi: 10.3390/molecules28031372 (PMC9920175; doi:10.3390/molecules28031372)
Supplement: Supplementary file 1 [file molecules-28-01372-s001.zip › molecules-2149737-supplementary.pdf]

SUPPLEMENTARY MATERIAL

Evaluation of hold-up volume determination methods and markers in hydrophilic interaction liquid chromatography

Lídia Redón, Xavier Subirats, Martí Rosés

Institute of Biomedicine (IBUB) and Department of Chemical Engineering and Analytical Chemistry, Universitat de Barcelona, Martí i Franquès 1-11, 08028 Barcelona, Spain

---

Dr. Xavier Subirats

Phone: +34 934 039 119, e-mail: xavier.subirats@ub.edu

Simultaneous contribution of HILIC and RPLC retention mechanisms

When the water content in the mobile phase is higher enough, for instance 50% of acetonitrile (Figure 1c) and 50% of methanol (Figure 1f), both retention trends can be clearly observed and U-shape curves are obtained. In this case, the data cannot be well fitted to Equation (6) or even to Equation (5) because both HILIC and RPLC mechanisms contribute simultaneously to retention. Bonded zwitterionic packing and some adsorbed water-rich layers act similarly as stationary phases. Then, the retention can be modelled by the sum of two different equations of the type of Equation (5), one with HILIC solvent descriptors and the other with RPLC solvent descriptors, which will lead to Equation (S1) for a unique homologous series assuming the same hold-up volume for both retention mechanisms:

$$V_R = V_M \left(1 + r^0_{HILIC} \cdot 10^{v_{HILIC} \cdot V} + r^0_{RPLC} \cdot 10^{v_{RPLC} \cdot V}\right) \quad (S1)$$

or Equation (S2) if several series are fitted altogether:

$$V_R = V_M \left(1 + \sum_{i=1}^{n} f_i \left(r^0_{HILIC,i} \cdot 10^{v_{HILIC} \cdot V} + r^0_{RPLC,i} \cdot 10^{v_{RPLC} \cdot V}\right)\right) \quad (S2)$$

The results obtained with this approach are presented in Table S2. They are less precise than the ones for most pure HILIC or RPLC regions (higher standard deviations of the fitting parameters), but quite reasonable [36, 52]. Retention in this region is a combination of HILIC and RPLC retention, which can be estimated by the calculated fitting parameters, each series r^0_{HILIC} and common v_{HILIC} in one hand, and each series r^0_{RPLC} and common v_{RPLC} on the other one, in addition to the constant V_M. Despite RPLC retention is more important for the largest solutes and HILIC retention for the lowest ones, both retention modes are significant for almost all solutes.

The polarity of high organic mobile phases is lower than that of the packing stationary phase, so the most polar components of the mobile phase are sorbed to form the HILIC stationary phase. This will be more polar and structured than the mobile phase and solutes will be retained by HILIC mechanism. At low concentrations of organic solvent, it will be the contrary. Water-rich mobile phases may be more polar and structured than the packing stationary phase, no significant water-rich sorbed HILIC layers can be formed, and thus only RPLC retention is possible. In this case, all solvent inside the column is expected to be mobile phase and the stationary phase is only the zwitterionic bonded phase. At intermediate organic solvent compositions, the polarity and structure of the mobile and packing phases are more similar and both retention mechanisms may act simultaneously [35, 51].

In fact, the homologous series method allows to classify retention as HILIC or RPLC (or mixed) according to the negative or positive v value. All members of a homologous series have the same hydrogen bond and polarity/polarizability properties (see Table S1). According to Equation (5), differences in the retention of the members of the series will depend only on their volumes. Creation of

a cavity in the most structured phase will be easier for the smaller solutes, whereas largest solutes will tend to be solvated by the less structured phase. Thus, in HILIC region, retention in the structured water-rich layers will decrease when the volume of the solute increases ($v < 0$). In RPLC, retention by the less structured packing phase is favored for the largest solutes and retention will increase with solute volume ($v > 0$). In the intermediate region, small solutes will be slightly retained by the remaining HILIC stationary phase, somewhat more polar than the mobile phase, whereas large solutes will be somewhat retained by the RPLC packing stationary phase, slightly less polar than the mobile phase. Retention variation is quite poor and U-shaped plots are obtained (a branch with $v < 0$ and the other with $v > 0$), as Figure 1c shows for acetonitrile/water mobile phase and Figure 1f for methanol/water.

Table S1

Molecular descriptors of the homologues considered in this work. For each homologous series the mean values ± standard deviation are provided for *E*, *S*, *A*, and *B* descriptors [39].

**Homologous series**	***E***	***S***	***A***	***B***	***V***
***n*-Alkyl benzenes**					
Benzene	0.61	0.52	0.00	0.14	0.716
Toluene	0.60	0.52	0.00	0.14	0.857
Ethylbenzene	0.61	0.51	0.00	0.15	0.998
Propylbenzene	0.60	0.50	0.00	0.15	1.139
Butylbenzene	0.60	0.51	0.00	0.15	1.280
Pentylbenzene	0.59	0.51	0.00	0.15	1.421
Hexylbenzene	0.59	0.50	0.00	0.15	1.562
Octylbenzene	0.58	0.48	0.00	0.15	1.844
Dodecylbenzene	0.57	0.47	0.00	0.15	2.407
Mean±SD	*0.59±0.01*	*0.50±0.02*	*0.00±0.00*	*0.15±0.00*	
***n*-Alkyl phenones**					
Acetophenone	0.82	1.01	0.00	0.48	1.014
Propiophenone	0.80	0.95	0.00	0.51	1.155
Butyrophenone	0.80	0.95	0.00	0.51	1.296
Valerophenone	0.80	0.95	0.00	0.50	1.437
Hexanophenone	0.78	0.95	0.00	0.51	1.578
Heptanophenone	0.77	0.95	0.00	0.50	1.718
Octanophenone	0.77	0.95	0.00	0.50	1.859
Nonanophenone	0.76	0.95	0.00	0.50	2.000
Decanophenone	0.75	0.95	0.00	0.50	2.141
Mean±SD	*0.78±0.02*	*0.96±0.02*	*0.00±0.00*	*0.50±0.01*	
***n*-Alkyl ketones**					
Propanone	0.18	0.70	0.04	0.49	0.547
Butanone	0.17	0.70	0.00	0.51	0.688
Pentan-2-one	0.14	0.68	0.00	0.51	0.829
Hexan-2-one	0.14	0.68	0.00	0.51	0.970
Heptan-2-one	0.12	0.68	0.00	0.51	1.111
Octan-2-one	0.11	0.68	0.00	0.51	1.252
Nonan-2-one	0.11	0.68	0.00	0.51	1.392
Decan-2-one	0.11	0.68	0.00	0.51	1.533
Undecan-2-one	0.10	0.68	0.00	0.51	1.674
Dodecan-2-one	0.10	0.68	0.00	0.51	1.815
Tridecan-2-one	0.10	0.68	0.00	0.51	1.956
Pentadecan-2-one	0.10	0.68	0.00	0.51	2.238
Nonadecan-2-one	0.09	0.68	0.00	0.51	2.801
Mean±SD	*0.12±0.03*	*0.68±0.01*	*0.00±0.01*	*0.51±0.01*	

Table S2

Hold-up volumes (V_M), volume coefficient (v), homologous series parameters (r^0 ± standard deviation) of the ZIC-HILIC column at the different mobile phase compositions obtained from the fittings of retention to Eqs. (6) and (S2). Overall statistics also given.

ϕ_{org} (v/v)	V_M (mL)	N	R^2_{adj}	$RMSE$	Behavior	v	$r^0_{benzenes}$	$r^0_{phenones}$	$r^0_{ketones}$
**Acetonitrile**									
**100%**	1.84±0.00	31	0.982	0.004	HILIC	-0.56±0.05	0.12±0.01	0.23±0.02	0.16±0.01
**90%**	1.71±0.00	31	0.985	0.004	HILIC	-0.67±0.04	0.11±0.01	0.23±0.02	0.23±0.01
**80%**	1.62±0.00	31	0.993	0.004	HILIC	-0.83±0.03	0.17±0.01	0.38±0.03	0.38±0.02
**70%**	1.55±0.00	29	0.996	0.003	HILIC	-0.82±0.03	0.22±0.01	0.49±0.03	0.50±0.02
**60%**	1.52±0.00	31	0.997	0.003	HILIC	-0.78±0.03	0.45±0.01	0.86±0.04	0.84±0.03
					RPLC	2.34±0.41	3.6E-7±8.7E-7	1.0E-13±4.0E-11	1.3E-8±3.5E-8
**50%**	1.51±0.01	31	0.999	0.002	HILIC	-0.64±0.03	0.51±0.01	0.81±0.03	0.77±0.02
					RPLC	1.88±0.06	1.3E-5±4.6E-6	2.2E-6±1.0E-6	3.4E-6±1.4E-6
**Methanol**									
**100%**	1.82±0.00	31	0.985	0.004	HILIC	-0.59±0.04	0.15±0.01	0.28±0.03	0.16±0.01
**90%**	1.83±0.00	31	0.987	0.004	HILIC	-0.62±0.04	0.16±0.01	0.25±0.02	0.16±0.01
**80%**	1.83±0.00	29	0.993	0.003	HILIC	-0.75±0.04	0.20±0.01	0.32±0.03	0.19±0.01
**70%**	1.83±0.00	27	0.995	0.003	HILIC	-0.83±0.04	0.26±0.01	0.40±0.03	0.24±0.01
**60%**	1.80±0.01	29	0.997	0.002	HILIC	-0.67±0.04	0.52±0.02	0.67±0.03	0.44±0.01
					RPLC	2.00±0.08	3.3E-5±1.2E-5	3.3E-6±1.5E-6	4.0E-6±1.8E-6
**50%**	1.79±0.03	28	0.998	0.008	HILIC	-0.54±0.12	0.59±0.07	0.67±0.10	0.44±0.03
					RPLC	2.44±0.06	3.6E-5±9.9E-6	2.1E-6±7.0E-7	2.4E-6±8.1E-7
